# Diagnostic algorithms for tuberculosis in Europe: insights from the European Reference Laboratory Network for Tuberculosis (ERLTB-Net)

**DOI:** 10.1016/j.lanepe.2025.101516

**Published:** 2025-11-15

**Authors:** Francesca Saluzzo, Andrea Maurizio Cabibbe, Richard Anthony, Alexandra Aubry, Francis Drobniewski, Yen Holicka, Troels Lillebaek, Rita Macedo, Mikael Mansjö, Viktoria Szél, Ljiljana Žmak, Daniela M. Cirillo, Ramona Groenheit

**Affiliations:** a Division of Immunology, Transplantation and Infectious Diseases, IRCCS San Raffaele Scientific Institute, 20132, Milan, Italy; b CRIMEDIM - Center for Research and Training in Disaster Medicine, Humanitarian Aid and Global Health, Università del Piemonte Orientale, 28100, Novara, Italy; c National Tuberculosis Reference Laboratory, IDS, National Institute for Public Health and the Environment (RIVM), Bilthoven, the Netherlands; d AP-HP. Sorbonne-Université, Centre National de Référence des Mycobactéries et de la Résistance des Mycobactéries aux Antituberculeux, Sorbonne Université, CNRS, Inserm, Centre d'Immunologie et des Maladies Infectieuses, CIMI, Paris, France; e Department of Infectious Disease, Imperial College London, London, UK; f International Reference Laboratory of Mycobacteriology, Statens Serum Institut, Copenhagen, Denmark; g Global Health Section, Department of Public Health, University of Copenhagen, Denmark; h National Tuberculosis Reference Laboratory, Department of Infectious Diseases, National Institute of Health Dr. Ricardo Jorge, Lisboa, Portugal; i National and WHO Supranational Reference Laboratory for Tuberculosis, Public Health Agency of Sweden, 171 82, Solna, Sweden; j National Reference Laboratory for Mycobacteriology, National Koranyi Institute for Pulmonology, 1121, Budapest, Hungary; k National Reference Laboratory for Tuberculosis, Croatian Institute of Public Health, Zagreb, Croatia

**Keywords:** Tuberculosis, Drug resistance, Diagnostic algorithms, Whole genome sequencing, EU/EEA

## Abstract

The reported poor treatment outcomes for extensively drug-resistant tuberculosis (TB) in the European region highlight the urgent need for effective and context-appropriate diagnostic strategies. While the World Health Organisation (WHO) provides model algorithms, these require adaptation to the European Union/European Economic Area (EU/EEA) context, a setting with low TB incidence but high resources. This viewpoint from the European Reference Laboratory Network for TB (ERLTB-Net) proposes a tailored diagnostic algorithm that prioritises the universal use of WHO-recommended molecular rapid diagnostic tests, systematic culture, and whole genome sequencing (WGS). This approach integrates phenotypic drug susceptibility testing strategically and outlines the possible role of targeted next-generation sequencing (tNGS) in the EU/EEA setting. The algorithm also addresses the importance of diagnostic harmonisation, cross-border collaboration, and sustained investment in sequencing capacity. By aligning diagnostic practices with the regional epidemiology and laboratory infrastructure, this stepwise, resource-sensitive approach aims to strengthen TB control, improve treatment outcomes, and guide public health action in the EU/EEA.

## Introduction

Poor treatment outcomes among individuals affected by extensively drug-resistant tuberculosis (TB) in the WHO European Region, as reported by Kherabi et al.^1^ underscore the urgent need for reliable and advanced diagnostic tools and algorithms, which are crucial for ensuring timely and appropriate treatment. Efficient diagnosis improves outcomes, reduces transmission, and helps prevent the development of drug resistance.

The World Health Organisation (WHO) has developed four model algorithms to optimise TB diagnosis and the detection of drug resistance, particularly multidrug- or rifampicin-resistant TB (MDR/RR-TB).^2^ Algorithm 1 serves as the starting point for initial TB diagnosis in all settings, prioritising the use of WHO-recommended molecular rapid diagnostics (mWRDs). Algorithm 2 integrates lipoarabinomannan (LAM) testing for TB diagnosis in people living with HIV (PLHIV). Algorithms 3 and 4 guide follow-up testing to detect additional drug resistance across all settings. Specifically, Algorithm 3 (3a/3b) identifies second-line resistance in RR/MDR-TB cases, while Algorithm 4 is designed to detect resistance in individuals with rifampicin-susceptible TB who are at risk of developing drug resistance.^3^

These algorithms function as a sequential cascade, emphasising universal access to rapid molecular testing as a prerequisite before incorporating other diagnostic methods. Nevertheless, challenges remain, particularly in balancing test specificity, pre-test probability, and clinical correlation. Effective implementation requires more than just the initial deployment of rapid molecular diagnostics; it also necessitates local adaptation and customisation. Key considerations include regional epidemiology, such as the prevalence of MDR/RR-TB, isoniazid monoresistance (HR), and HIV, along with the testing volumes and the availability of referral systems, resources, infrastructures, and technical expertise.^4^, ^5^, ^6^

In this viewpoint we outline a TB diagnostic algorithm that considers the specific characteristics and variations in TB diagnostic strategies within the European Union/European Economic Area (EU/EEA) focusing on the initial use of rapid molecular testing but also the role of conventional culture methods and WGS. In doing so, the proposed algorithm acknowledges that the EU/EEA represents a relatively high-resource, low TB burden setting, which influences the pre-test probability and, consequently, the positive predictive value of diagnostic tests.

## Why a tailored diagnostic algorithm is needed in the EU/EEA

The EU/EEA includes diverse geographic and epidemiologic contexts and at-risk populations, including migrants from high TB burden countries, the elderly, and immunosuppressed individuals (such as those living with HIV or undergoing immunosuppressive treatment), who are disproportionately affected by TB.^7^ Although the region is predominantly low incidence (38,993 reported cases, 8.6 cases per 100,000 population in 2023), it reports a relatively high proportion of RR/MDR TB, particularly in high-risk groups and specific countries.^8^

This presents a paradox. On the one hand, lower overall TB incidence reduces the need for infrastructure and laboratory networks, as well as clinical familiarity and experience in the management of this disease. On the other hand, the challenge of drug resistance (DR) and free movement within the EU/EEA and to adjacent countries facilitates the spread of infectious diseases.^9^ This makes standardised diagnostic algorithms essential for early and accurate detection, DR prediction, effective treatment, tracking transmission, including cross-border cases, and timely public health interventions. Harmonisation also fosters collaboration, mutual support, and data sharing, which are crucial for managing complex cases, particularly those involving RR/MDR-TB.

EU/EEA countries reported that, in 2023, 80% of bacteriologically confirmed pulmonary TB cases were tested for at least rifampicin resistance and that over 70% had drug susceptibility testing (DST) results for first-line drugs, denoting stable but still incomplete access to universal DST. Among the notified cases with RR/MDR-TB, 60% were tested for fluoroquinolone resistance, and almost all pre-extensively drug resistant (pre-XDR) cases had DST results reported for at least one other Group A drug.^8^ Nonetheless, significant differences between the countries were observed, and poor clinical outcomes, especially for XDR-TB, associated with the current testing strategies, call for a standardised approach allowing for prompt identification of TB and drug resistance.

In August 2024, the European Reference Laboratory Network for Tuberculosis (ERLTB-Net) conducted a survey to assess TB diagnostic practices and algorithms implemented across EU/EEA countries. The survey received responses from EU/EEA National Reference Laboratories (NRLs) representing 23 countries.

The survey shows that adoption of the WHO's four model diagnostic algorithms^2^ varies across reporting countries. Most countries (22/23) use a mWRD and smear microscopy as primary diagnostics. Moreover, for the early detection of resistance to first-line drugs, particularly rifampicin and isoniazid, line probe assays (LPAs) and moderate-complexity nucleic acid amplification tests (NAATs) are used, respectively, in 14/23 and 17/23 countries as initial tests. Culture is universally performed to confirm TB diagnosis. Phenotypic drug susceptibility testing (pDST) for first-line drugs is performed in all countries for all *Mycobacterium tuberculosis* complex (MTBC) isolates, except for two laboratories that report performing first-line DST only if mutations conferring resistance are identified through WGS. Second-line pDST is typically reserved for selected cases.

Genotypic DST (gDST) is mainly performed at the central or national level, with thirteen countries systematically using WGS and two employing targeted NGS. Previous publications from the group report the increased availability of WGS in the EU/EEA.^10^ Moreover, in 2024, 20/33 EU/EEA NRLs participated in the external quality assessment (EQA) round for MTBC genotyping (NGS) organised by the ERLTB-Net, marking a continuous increase in numbers, as only 13 NRLs participated in the first EQA round in 2017.^10^

These findings reveal variability in advanced molecular testing and algorithm implementation, underscoring the need for continued harmonisation and capacity building across the region. The wide availability of liquid-media based microbiological culture and WGS underline that most EU/EEA laboratories require a tailored algorithm to avoid duplication of tests and overspending on TB diagnostics.^11^

The rising prevalence of opportunistic non-tuberculous mycobacteria (NTM), reported for example in Denmark, France and Germany, which often mimic TB clinically or radiologically, further complicates diagnosis.^12^, ^13^, ^14^ Microbiological culture (associated with microscopy) ensures that these cases are not missed and potentially diagnosed earlier. In this context, awareness of local mycobacteria epidemiology represents a pivotal element to guide implementation of the most appropriate diagnostics strategy, and this should be supported by an algorithm tailored to fulfil this scope.

## The EU/EEA algorithm

**Fig. 1:**
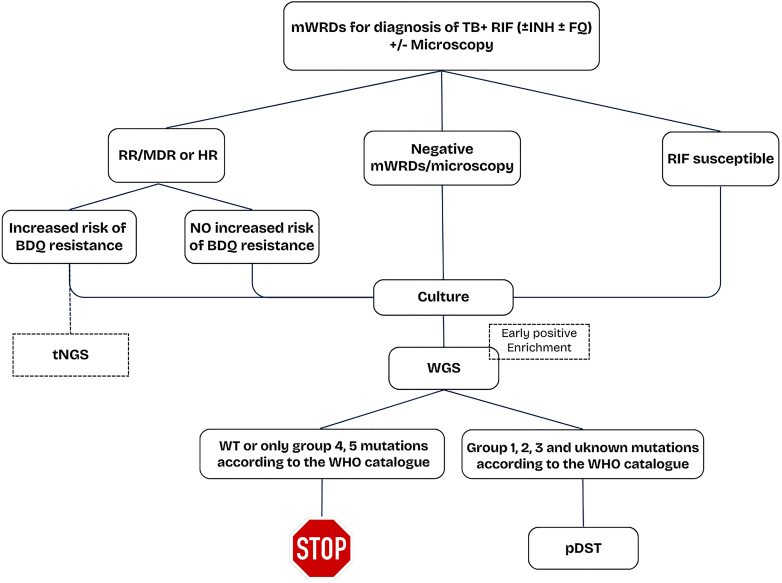
**Diagnostic algorithm for tuberculosis (TB) and drug resistance detection in the EU/EEA.** Initial testing uses WHO-recommended molecular rapid diagnostic tests (mWRDs) to detect TB and rifampicin resistance, with optional assessment of isoniazid and fluoroquinolone resistance. Confirmed cases proceed to culture, whole genome sequencing (WGS), and phenotypic drug susceptibility testing (pDST). WGS enables genotypic DST (gDST), guides treatment adjustments for drug-susceptible (DS) and drug-resistant (DR) TB and supports outbreak investigation and epidemiological surveillance. pDST is performed when MTBC mutations associated with resistance (group 1–2) or of uncertain significance (group 3) are detected, or when mutations are not listed in the WHO catalogue, allowing confirmation of gDST results, treatment adjustment, and evaluation of resistance to new or repurposed compounds. Targeted next-generation sequencing (tNGS) serves as a backup for gDST, enabling rapid initiation or modification of RR/MDR- and HR-TB regimens when culture is unavailable, contaminated, or WGS capacity is limited. Smear microscopy complements the pathway by assessing infectiousness and helps to initially identify non-tuberculous mycobacteria (NTM) in cases of negative mWRDs which are definitively detected through microbiological culture and subsequent identification. *Acronyms: FQ: fluoroquinolones, INH: isoniazid, RIF: rifampicin, HR: isoniazid resistant, MDR: multidrug resistant, RR: rifampicin resistant, S: susceptible*.

Considering the specific characteristics of the EU/EEA context, the diagnostic algorithm proposed in Fig. 1 prioritises the universal use of mWRDs for the initial detection of MTBC and rapid screening of resistance to at least rifampicin (and preferably isoniazid and fluoroquinolones, depending on local epidemiology). Furthermore, regardless of the initial diagnostic outcome—whether TB is confirmed or not—and irrespective of the rifampicin resistance status, culture is systematically performed.

While aligned with WHO Algorithm 1, which prioritises the use of mWRDs for initial TB diagnosis,^3^ this approach leverages the available resources in the EU/EEA to enhance diagnostic breadth. The universal use of culture ensures the availability of isolates for downstream phenotypic and genotypic testing, maximising the diagnostic yield for both MTBC and NTMs.^12^ In this context, smear microscopy might also be performed primarily as a marker of TB infectiousness and to evaluate the need for respiratory isolation. Moreover, microscopy can support the evaluation of NTM infection when positive, but mWRDs for MTBC are negative^15^ and to monitor treatment response at regular intervals.

In consideration of the increased availability of next-generation sequencing (NGS) technologies in the EU/EAA,^10^ following a positive culture, WGS shall be systematically used to generate genotypic DST (gDST) results. These results can support treatment adjustments informed by mWRDs for both drug-susceptible and drug-resistant TB, particularly RR/MDR and HR cases, which require comprehensive DST data.^16^^,^^17^ Moreover, in a context where migration from high-burden countries is common,^9^ WGS plays a crucial role in identifying cross-border TB clusters, tracking transmission dynamics, and monitoring the spread of DR strains.^18^^,^^19^ As demonstrated by the ECDC pilot study EUSeqMyTB, by identifying the genetic relatedness between cases, WGS strengthens epidemiological surveillance, supports contact tracing, and enables targeted, coordinated public health interventions across regions and borders.^11^ Further studies have demonstrated the value of systematic implementation of WGS from both clinical and public health perspectives. It has been demonstrated that WGS in low burden countries can reduce turnaround time compared with pDST, while providing comprehensive drug susceptibility information, and enabling outbreak investigation and surveillance.^20^, ^21^, ^22^ In the United Kingdom, WGS has been integrated into the national TB program, allowing timely detection of drug resistance from bacteriological cultures which inform treatment decisions.^23^ In this context it has been demonstrated that WGS shortens the time to drug sensitivity testing and treatment modification when needed potentially resulting in a reduction in treatment costs.^23^ The study by Mugwagwa et al. also demonstrated how the maximum cost effectiveness of WGS is reached when it is coupled with mWRDs.^23^ Furthermore, even taking into account the cost of WGS, evidence from multiple cost-effectiveness analyses in low-burden, high-resource settings now supports the programmatic use of WGS for drug resistance detection and transmission analyses.^23^^,^^24^ To further reduce turnaround time and considering the rapid developments in NGS technologies, WGS may be applied to early positive cultures (i.e. those showing initial visible growth after short incubation periods) or, in selected cases, directly to clinical specimens. However, this approach requires standardised and optimised procedures, including automated workflows for MTBC DNA extraction and purification.^25^^,^^26^ An alternative approach, likely suitable for respiratory samples with high bacterial load, is direct sequencing using whole-genome hybridization enrichment, which holds promise for further improving efficiency.^27^^,^^28^

In the suggested approach, phenotypic drug susceptibility testing (pDST), performed after culture positivity, serves as a complementary approach to gDST. It confirms resistance profiles or susceptibility, builds the knowledge base to identify new mutations associated with resistance and guides treatment modifications, particularly for new and repurposed compounds.

Therefore, according to the suggested algorithm, pDST is reserved for cases where WGS identifies MTBC mutations associated with resistance (Group 1 and 2, i.e. including associated with resistance and interim association), mutations of uncertain significance (Group 3), as well as mutations not covered by drug-specific grading rules or non-synonymous mutations not yet included in the WHO catalogue of mutations, particularly for key group A, B and C drugs.^29^^,^^30^ Mutations of unknown or uncertain significance are initially interpreted as susceptible, pending phenotypic confirmation. In this algorithm, pDST is not performed to confirm gDST-susceptible results for first-line drugs. This ensures that pDST is applied strategically, mainly to address the suboptimal sensitivity of gDST for specific drugs. This well aligns with ongoing approaches and studies aimed at potentially eliminating the need for pDST in routine tuberculosis diagnosis.^20^^,^^31^^,^^32^

Currently three targeted next-generation sequencing (tNGS) solutions are recommended by the WHO as follow-on tests to be performed directly from clinical specimens for additional resistance detection.^3^ Developers are actively expanding these platforms to include all key drugs used in RR/MDR regimens. In the EU/EEA context, when culture is unavailable, contaminated, or delayed, or when WGS capacity is limited due to lack of bioinformatics capacity, tNGS may serve as a backup solution for comprehensive gDST and rapid initiation or adjustment of RR/MDR-TB and HR-TB treatment regimens, acknowledging suboptimal sensitivity for new/repurposed drugs. Moreover, WHO-approved tNGS platforms could be particularly valuable in cases of high risk of bedaquiline (BDQ) resistance (e.g., contact of BDQ-R, previous BDQ-based treatment failure, high prevalence of BDQ-R in the country). Nonetheless, considering the current limitations of these platforms, pDST should be performed systematically.

## Conclusions

This tailored EU/EEA algorithm differs from the WHO ones as it practically addresses regional epidemiology while considering laboratory capacity and the increasing availability of WGS in this geographical area. While the WHO algorithms have been designed for addressing the global TB burden focusing on the use of mWRDs in low-resource settings, this EU/EEA approach leverages laboratory capacity and a strategic combination of culture, WGS, and selective phenotypic DST to optimise diagnostic efficiency and rapidly detect both TB and drug resistance. By carefully integrating the available tools, the proposed algorithm ensures that MTBC isolates are available for genotypic DST and transmission cluster analyses, enabling the monitoring of cross-border transmission and emerging resistance patterns.

Therefore, this tailored approach might enable not only more prompt identification of resistance, supporting the clinical management of people with TB, but also timely public health interventions through standardised data sharing and coordinated outbreak management. Strengthening TB diagnostic capacity in the EU/EEA is therefore essential, particularly considering rapid technological progress, emerging drug resistance, lower success rate of DR-TB treatment,^1^ and ongoing migration from high-burden countries.^33^ Since 2009, the ERLTB-Net has supported the enhancement of TB laboratory capacity across the region through structured training initiatives, raising awareness of TB and MDR-TB among healthcare professionals.^12^^,^^34^ Over the past five years, these efforts have increasingly focused on NGS and pDST, in line with the evolving diagnostic landscape and the needs of the EU/EEA laboratory network. This stepwise, integrated algorithm ensures robust, harmonised TB diagnosis and resistance detection across EU/EEA countries and laboratory networks, while maintaining adaptability to local epidemiological context and laboratory capacity. Pairing this approach with close collaboration with public health authorities and policy makers will ensure that advances in diagnostic capacity translate into improved case detection and outcomes.

## Contributors

Francesca Saluzzo: Conceptualisation, Writing—Original Draft, Writing—Review & Editing; Andrea Maurizio Cabibbe: Conceptualisation, Writing—Original Draft, Writing—Review & Editing; Richard Anthony: Conceptualisation, Writing—Review & Editing; Alexandra Aubry: Writing—Review & Editing; Francis Drobniewski: Conceptualization, Writing—Review & Editing; Yen Holicka: Project Administration, Writing—Review & Editing; Troels Lillebaek: Writing—Review & Editing; Rita Macedo: Writing—Review & Editing; Mikael Mansjö: Writing—Review & Editing; Viktoria Szél: Writing—Review & Editing; Ljiljana Žmak: Writing—Review & Editing; Daniela M. Cirillo: Conceptualisation, Supervision, Funding Acquisition, Writing—Review & Editing Ramona Groenheit: Conceptualisation, Writing—Review & Editing, Validation.

## Declaration of interests

AA's research team received Grants from (i) Janssen and Bioversys for research purpose in the frame of European projects (IMI H2020) and TB Alliance for PTM resistance surveillance. RA funded by ECDC to attend ERLTB-Net meetings as country representative. Travel and accommodation costs only, no payment. VS acknowledges funding from the European Centre for Disease Prevention and Control (ECDC) for the ERLTB-Net-3 project through ECDC/GRANT/2022/010 grant.

All other authors have no conflict of interest.
